# Factors influencing the presence of *Candida dubliniensis* and other *non-albicans* species in patients with oral lichen planus: a retrospective observational study

**DOI:** 10.1007/s00784-021-04004-5

**Published:** 2021-06-18

**Authors:** Florian Molkenthin, Moritz Hertel, Konrad Neumann, Andrea Maria Schmidt-Westhausen

**Affiliations:** 1 CharitéCentre 3, Department of Periodontology, Oral Medicine and Oral Surgery, Charité - Universitätsmedizin Berlin, Aßmannshauser Str. 4, 14197 Berlin, Germany; 2 Institute of Medical Biometrics and Clinical Epidemiology, Charité – Universitätsmedizin Berlin, Charitéplatz 1, 10117 Berlin, Germany

**Keywords:** *Candida*, Oral lichen planus, *Non-albicans* species, Oral candidiasis

## Abstract

**Objectives:**

The epidemiologic distribution of *non-albicans* species in the oral cavity of oral lichen planus (OLP) patients remains uncertain. Therefore, the aim of this study was to identify factors associated with the presence of *C. dubliniensis* and other *non-albicans* species. Furthermore, independent risk factors for *Candida* superinfection in OLP should be identified.

**Material and methods:**

Epidemiologic data and microbiological findings from 268 symptomatic OLP patients who underwent continuous oral swab culture over a 5-year period (2015–2019) were retrospectively reviewed. *Candida* species identification and semi-quantification were obtained by culture on CHROMagar Candida, followed by matrix-assisted laser desorption/ionization time-of-flight mass spectrometry (MALDI-TOF MS).

**Results:**

*C. albicans* was the most frequently isolated species (72.3%), followed by *C. glabrata* (7.3%), *C. dubliniensis* (5.8%), *C. krusei* and *C. parapsilosis* (both 2.6%). The presence of *C. dubliniensis* was significantly associated with tobacco smoking. Other *non-albicans* spp. were significantly more often detected in patients using removable dentures. Increasing age and the intake of psychotropic drugs were identified as independent risk factors of *Candida* superinfection in OLP.

**Conclusion:**

In OLP patients, certain local and systemic factors increase the risk of carrying potentially drug-resistant *Candida* species and the development of *Candida* superinfection of OLP lesions.

**Clinical relevance:**

Due to the frequent detection of *non-albicans* species in OLP, resistance or at least reduced sensitivity to azole antifungals should be expected, especially in smokers and patients using removable dentures. In the case of oral complaints, a superinfection with *Candida* should be considered, whereby older patients and patients taking psychotropic drugs have an increased risk for oral infection with *Candida*.

## Introduction

Due to the frequent and preventive use of antifungal drugs in immunocompromised patients and due to more precise and faster methods for the identification of *Candida* species, the emergence of *non-albicans* species (spp.) has been observed in recent years [1]. Although these species typically lack the range of virulence factors found in *C. albicans*, they have come to prominence due to their frequent intrinsic or acquired resistance to azole antifungals [2, 3]. A paradigm of this selective process could be the emergence of *C. dubliniensis* [4]. This species shares many phenotypic characteristics with *C. albicans*, including the ability to produce hyphae and chlamydospores, features previously associated only with *C*. *albicans* [5]. Despite the phenotypic similarities, it is less pathogenic and less frequently isolated from the oral cavity [6]. However, *C. dubliniensis* is increasing in clinical significance as this species is potentially less susceptible or resistant to fluconazole, although mostly after extensive prophylaxis/therapy with this drug [7]. Furthermore, it has been demonstrated that fluconazole resistance in *C. dubliniensis* is easily inducible in vitro and that the adherence of this organism to epithelial cells increases in the presence of fluconazole [8, 9].

Oral mucosal diseases, such as oral lichen planus (OLP), may be influenced by the presence of *Candida* spp. [10–12]. OLP is a chronic inflammatory disease of autoimmune origin that affects between 0.9 and 2.6% of the population [13]. Several *Candida* species have been isolated from the oral cavity of OLP patients, with *C. albicans* being the most common [12, 14, 15]. However, it has been shown that *non-albicans* species are more frequently present in OLP patients compared to healthy subjects [16].

A challenge in the treatment of OLP is the presence or development of *Candida* superinfection, which makes antimycotic therapy necessary. Infections with *Candida* species can lead to both exacerbation and obscuration of the clinical features of OLP, as both conditions may be associated with burning pain and erythematous lesions [17]. In addition, hyphal invasion is a potential risk factor for malignant transformation through the production of carcinogenic metabolites of *Candida* such as nitrosamines and acetaldehyde [18, 19].

The presence of *non-albicans* species in patients with oral mucosal alterations and the respective clinical impact, especially of *C. dubliniensis*, remain partly unknown. In order to initiate an adequate antifungal therapy in OLP patients while avoiding potential problems related to microbial resistance, the aim of this study was *(i)* to determine factors associated with the presence of *C. dubliniensis* and other *non-albicans* species and *(ii)* to identify independent risk factors for the presence of *Candida* superinfection of OLP lesions.

## Material and methods

### Study design

A retrospective, observational and cross-sectional study was conducted at the Department of Periodontology, Oral Medicine and Oral Surgery of the Charité – Medical University Berlin. Microbiological findings and records of symptomatic OLP patients treated at the department between 01 January 2015 and 31 December 2019 (5 years) were eligible.

All patients (i) with the clinical and/or histological diagnosis of oral lichen planus (ii) and who underwent an oral swab culture testing for the presence of *Candida* spp. were included. The clinical diagnostic criteria for OLP included the presence of bilateral, more or less symmetrical lesions, the presence of a reticular pattern with grayish-white lines (*Wickham* striae) and papular, plaque-like, erythematous, erosive and bullous variants as subtypes. Exclusion criteria comprised all patients (i) with clinical/histological mucogingival disease other than OLP, (ii) under antifungal (including chlorhexidine) or antibiotic therapy, (iii) under systemic immunosuppressive therapy, human immunodeficiency virus (HIV)-positive patients and (iv) patients with a history of anaemia, and radiation therapy or surgery for head and neck malignancy.

During the above-mentioned period, all patients underwent an intraoral examination by an oral medicine specialist. Demographic data including age and gender, clinical features of OLP, current use of topical immunosuppressive drugs, concomitant diseases, medication taken, presence of removable dentures and complete smoking history (i.e. daily consumption and pack years) were documented. The data were recorded using a standardized data entry form. Based on this information, a de-identified database was created.

### Diagnosis of oral candidiasis

Superinfection with *Candida* species was diagnosed based on clinical signs (loss of lingual papillae, redness and fissures on the tongue, erythematous oral mucosa and white, removable plaques) and/or symptoms (loss of taste, burning, pain) associated with at least moderate growth of one *Candida* species in culture. Accordingly, these patients received local antifungal therapy with nystatin ointment or amphotericin B troches for at least 3 weeks.

### Yeast sampling and culture

Specimens were obtained by swabbing of the affected OLP lesions with a nylon-flocked sample collection swab (ESwab™, COPAN Diagnostics Inc., USA). After the swab was taken, the samples were immediately transferred into a transport tube with 1 ml Amies medium (COPAN Diagnostics Inc., USA) and sent to the laboratory within 24 h for microbiological diagnostics. Samples were fractionally spread on the culture media. Each sample was plated on Sabouraud Dextrose Agar (SDA with chloramphenicol and gentamicin, pH = 6.8 ± 0.2) and on CHROMagar™ Candida (Becton Dickinson, USA). The SDA plate was incubated at 28 °C, and the CHROMagar™ plate at 36 °C for 4 days each. If no growth occurred after 4 days, the incubation time was extended to 7 days. A presumptive assignment according to the growth on CHROMagar™ Candida was followed by the exact species identification. Samples from pre-cultured single colonies were analyzed by matrix-assisted laser desorption/ionization and time-of-flight mass spectrometry (MALDITOF MS, Vitek© MS, bioMérieux, France) or by biochemical identification with the Vitek© 2 ID-card (bioMérieux, France). In order to distinguish between *C. albicans* and *C. dubliniensis* the latex agglutination test (BICHRO-DUBLI FUMOUZE©, Biosynex, France) was performed after growth of green colonies on CHROMagar™ Candida. A semi-quantitative estimation of fungal growth was carried out, and the following classification was used: sporadic growth: ≤ 10 colony forming units (CFU) in the first section; low growth: > 10 CFU and growth in the first section; moderate growth: growth up to the second section; abundant growth: growth up to the third section.

### Statistical analysis

For all categorical variables absolute and relative frequencies were given. Continuous variables were described by mean value and standard deviation. Furthermore, Pearson’s Chi-square test (or Fisher’s exact test if one of the expected cell frequencies was below 5) was used to determine whether patient characteristics were associated with the detection of *C. dubliniensis* or other *non-albicans* species, and with the presence of *Candida* superinfection. Mean values of continuous variables were compared using Student’s *t*-test. All variables with *p*-values ≤ 0.2 in the simple bivariate analysis, including demographic, local, systemic and lifestyle factors, were included in binomial logistic regression models. We did not apply model selection algorithms such as backward or forward selection. The dependent variables of three logistic regression models were the presence of *C. dubliniensis* and *non-albicans* species, respectively, and the occurrence of *Candida* superinfection of OLP.

All statistical tests were performed two-sided and a *p*-value of ≤ 0.05 was considered significant. In this exploratory study, all *p*-values are unadjusted. Statistical analysis was performed using IBM® SPSS® Version 25.0.

## Results

### Study population

In total, 268 patients met the inclusion and exclusion criteria (210 females, mean age: 64.9 ± 11.7 years; 58 males, mean age: 60.2 ± 13.4 years). Demographic, clinical, and anamnestic characteristics of the patients are presented in Table 1. The demographic data and data regarding the clinical characteristics and therapy of OLP were completely available in all patients. Data on concomitant diseases were missing in two patients and data on medication, prosthetics and smoking history were missing in three patients, respectively. The mean age of the patients was 63.9 ± 12.2 years (range: 26–88), with the mean age of female patients being significantly higher than that of male patients (*p* = 0.019). In addition, female patients suffered significantly more frequently from hypothyroidism (*p* < 0.001), autoimmune diseases (except lichen planus; *p* = 0.025) and asthma/COPD (*p* = 0.047) compared to male patients. Regarding the clinical features of OLP, 112 patients (41.8%) showed erosive or ulcerative lesions at the time of examination. In addition, 33 patients (12.3%) had extraoral manifestations of lichen planus, while women were significantly more likely to have extraoral involvement (*p* = 0.020). In 142 of 268 cases (53.0%), a biopsy was taken, and the clinical diagnosis was confirmed by histopathological examination. In all other cases, a biopsy was not performed due to the typical pattern of OLP. Within the last 4 weeks before the examination, 42 of 268 patients (15.7%) applied topical steroids for a maximum of 14 consecutive days. Regarding drug history, 140 of 265 patients (52.8%) were treated with antihypertensives (ACE inhibitors, AT2 receptor blockers, beta-receptor blockers, diuretics and calcium antagonists). Among them, 57 patients (40.7%) took two or more antihypertensive drugs. Thirty-one of 265 patients (11.7%) regularly took psychotropic drugs (antidepressants, antipsychotics, antiepileptic drugs, anxiolytics, and hypnotics), with antidepressants being the most frequent (80.6%).

**Table 1:** Demographic, clinical and anamnestic characteristics of OLP patients

Feature (*n*)	Total	Female	Male	*P *^a^
	No. (%)	No. (%)	No. (%)	
Total	268 (100)	210 (78.4)	58 (21.6)	*–*
Mean age (SD)	63.9 (± 12.2)	64.9 (± 11.7)	60.2 (± 13.4)	0.019
Clinical features (268)				
Erosive	112 (41.8)	88 (41.9)	24 (41.4)	0.943
Non-erosive	156 (58.2)	122 (58.1)	34 (58.6)	
Extraoral involvement	33 (12.3)	31 (14.8)	2 (3.4)	0.020
Treatment (268)				
Topical steroids	42 (15.7)	37 (17.6)	5 (8.6)	0.095
Removable denture (265)				
Present	63 (23.8)	51 (24.6)	12 (20.7)	0.532
Concomitant disease (266)				
Hypertension	149 (56.0)	119 (57.2)	30 (51.7)	0.457
Hypothyroidism	76 (28.6)	72 (34.6)	4 (6.9)	< 0.001
Diabetes mellitus	36 (13.5)	29 (13.9)	7 (12.1)	0.712
Autoimmune disease	44 (16.5)	40 (19.2)	4 (6.9)	0.025
Asthma/COPD	28 (10.5)	26 (12.5)	2 (3.4)	0.047
Mental disorders	30 (11.3)	21 (10.1)	9 (15.5)	0.299
Allergies	88 (33.1)	71 (34.1)	17 (29.3)	0.490
Medication (265)				
Antihypertensives	140 (52.8)	111 (53.6)	29 (50.0)	0.625
NSAID	41 (15.5)	33 (15.9)	8 (13.8)	0.689
Inhaled steroids	14 (5.3)	12 (5.8)	2 (3.4)	0.741
Psychotropic drugs	31 (11.7)	23 (11.1)	8 (13.8)	0.574
Proton pump inhibitors	26 (9.8)	22 (10.6)	4 (6.9)	0.398
Lifestyle (265)				
Smoker	40 (15.1)	33 (15.9)	7 (12.1)	0.466
Former smoker	55 (20.8)	44 (21.3)	11 (19.0)	0.704

Complete data for concomitant diseases were available from 266 patients and for medication, presence of removable dentures, and smoking from 265 patients

*SD* standard deviation; *COPD* chronic obstructive pulmonary disease; *NSAID* non-steroidal anti-inflammatory drugs

^a ^Referring to inter-gender comparison

### Microbiological results

**Fig. 1:**
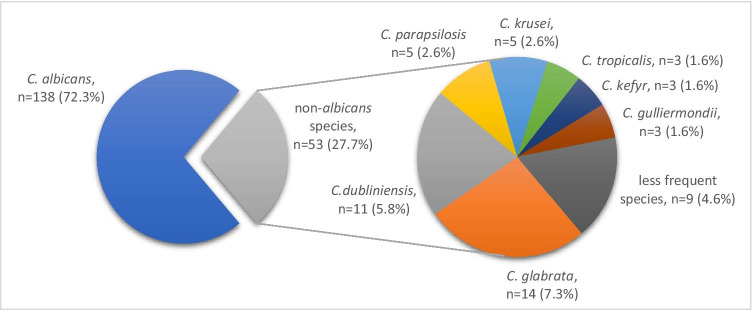
Distribution of *Candida* isolates from the oral cavity of OLP patients

In 160 of 268 OLP patients (59.7%), a total of 191 yeasts were isolated. The spectrum of isolated *Candida* species is shown in Fig. 1. Among all 191 isolates, *C. albicans* was the most frequent species (72.3%). Thirteen different *non-albicans* species accounted for 27.7%. The most common *non-albicans* species were *C. glabrata* (7.3%), *C. dubliniensis* (5.8%), *C. krusei* and *C. parapsilosis* (both 2.6%). Species known to be less susceptible or resistant to azole antifungals (*C. glabrata*, *C. krusei* and *C. dubliniensis*) accounted for 15.7% (30/191) of all isolates and 56.6% (30/53) of *non-albicans* species. In 22 samples, more than one *Candida* species was found (mixed cultures). *C. albicans* was isolated as monoculture in 117 patients. *C. dubliniensis* was isolated from oral samples of 11 patients, in 7/11 cases as monoculture and in 4/11 cases co-isolated as mixed culture with *C. albicans*. In 36 patients *non-albicans* species other than *C. dubliniensis* were isolated, with 16/36 cases being mixed cultures consisting of more than one species (with or without *C. albicans*).

### Factors associated with the presence of C. dubliniensis

Cross-tabulation analysis revealed that the presence of *C. dubliniensis* was not significantly associated with gender, clinical characteristics of OLP, current use of topical steroids, the presence of removable dentures, diabetes mellitus, other autoimmune diseases, use of antihypertensive drugs, psychotropic drugs, inhaled steroids or proton pump inhibitors. The mean age of patients with *C. dubliniensis* was significantly lower than that of patients with *C. albicans* (*p* = 0.039). Furthermore, tobacco smoking was significantly associated with colonization/infection by *C. dubliniensis* (*p* < 0.001) (Table 2).

**Table 2:** Comparison of factors influencing the detection of certain *Candida* species

Influencing factors	*Candida albicans* (*n* = 117)	*Candida dubliniensis* (*n* = 11)	*P *^a^	Other *non-albicans* spp. (*n* = 36)	*P *^a^
	No. (%)	No. (%)		No. (%)	
Demography					
Mean age (SD)	66.2 (± 10.5)	58.9 (± 16.1)	0.039	69.1 (± 9.5)	0.129
Gender (male)	24 (20.5)	0 (0)	–	6 (16.7)	0.611
Local					
Erosive OLP	56 (47.9)	4 (36.4)	0.465	13 (36.1)	0.215
Topical steroids	18 (15.4)	2 (18.2)	0.682	7 (19.4)	0.565
Inhaled steroids	6 (5.2)	2 (18.2)	0.145	2 (5.6)	1.000
Removable denture	28 (24.3)	4 (36.4)	0.469	21 (58.3)	< 0.001
Systemic					
Hypothyroidism	38 (32.8)	3 (27.3)	1.000	9 (25.0)	0.379
Diabetes mellitus	21 (18.1)	0 (0)	–	7 (19.4)	0.856
Autoimmune disease	20 (17.2)	3 (27.3)	0.418	11 (30.6)	0.083
Asthma/COPD	13 (11.2)	3 (27.3)	0.144	3 (8.3)	0.763
Drug-induced ^b^					
Antihypertensives	70 (60.9)	5 (45.5)	0.320	20 (55.6)	0.571
Psychotropic drugs	18 (15.7)	2 (18.2)	0.686	6 (16.7)	0.884
Proton pump inhibitors	13 (11.3)	1 (9.1)	1.000	6 (16.7)	0.397
Lifestyle					
Smoker	14 (12.2)	7 (63.6)	< 0.001	2 (5.6)	0.360
Former smoker	27 (23.5)	1 (9.1)	0.453	8 (22.2)	0.876

Classification: *C. albicans* monoculture, *C. dubliniensis*, and other *non-albicans* species (both with and without *C. albicans*). Complete data for concomitant diseases were available from 116 patients and for medication, presence of a removable denture and smoking from 115 patients with *C. albicans*

*SD* standard deviation; *OLP* oral lichen planus; *COPD* chronic obstructive pulmonary disease

^a ^Colonisation/infection with *C. albicans* as reference

^b ^Drugs with a high level of evidence for causing hyposalivation

A logistic regression model, including age, asthma/COPD, inhalative steroids and tobacco smoking showed that only tobacco smoking significantly increased the odds to carry *C. dubliniensis* (*p* = 0.001, OR = 10.51 (95% CI [2.52, 43.83]). The logistic regression model was statistically significant, *χ*^2^(4) = 18.13, *p* = 0.001. The Hosmer–Lemeshow test indicated a good model fit (*χ*^2^(7) = 4.99, *p* = 0.662). None of the correlations between predictor variables were high (Pearson correlation *r* < 0.70), indicating that multicollinearity was not a confounding factor in the analysis.

### Factors associated with the presence of other non-albicans species

Cross-tabulation and *t*-test analyses revealed no statistically significant associations between sex, age, clinical forms of OLP, current use of topical steroids, diabetes mellitus, other autoimmune diseases, use of antihypertensive drugs, psychotropic drugs, inhaled steroids or proton pump inhibitors, and smoking with the presence of *non-albicans* species other than *C. dubliniensis*. Compared to *C. albicans*, patients who wore a removable denture were significantly prone to colonization/infection by other *non-albicans* species (with or without *C. albicans*) (*p* < 0.001). Patients with other autoimmune diseases (than lichen planus) also tended to be more frequently colonized/infected with other *non-albicans* species, although this was not statistically significant (*p* = 0.083) (Table 2).

The binomial logistic regression analysis, including age, autoimmune diseases and removable dentures confirmed, that only the presence of removable dentures was statistically significant (*p* = 0.001), increasing the likelihood for the presence of *non-albicans* species other than *C. dubliniensis* in OLP patients (OR = 4.19, 95% CI [1.78, 9.88]). The logistic regression model was statistically significant, *χ*^2^(3) = 15.92, *p* = 0.001. The Hosmer–Lemeshow test indicated a good model fit (*χ*^2^(8) = 1.61, *p* = 0.991). Correlations between predictor variables were low (Pearson correlation *r* < 0.40), indicating that multicollinearity was not a confounding factor in the analysis.

### Risk factors for Candida superinfection

In total, in 97/268 patients (36.2%), superinfection of OLP with *Candida* species was diagnosed. Contingency table analysis showed no significant association of sex, current use of topical steroids, intake of proton pump inhibitors, inhaled steroids and positive smoking history with the occurrence of a *Candida* superinfection. The mean age of *Candida* superinfected patients was significantly higher than that of non-infected patients (*p* < 0.001). A statistically significant association was also found for the presence of removable dentures (*p* = 0.031), other autoimmune diseases (*p* = 0.041), the use of antihypertensive (*p* = 0.002) and psychotropic drugs (*p* = 0.002). Moreover, a significantly larger proportion of patients with erosive OLP than with non-erosive OLP suffered from *Candida* superinfection (*p* = 0.029). However, it was not possible to distinguish whether the erosive areas were the manifestation of lichen planus itself or the result of *Candida* infection. Therefore, this factor was not included into multivariate analysis. Patients with diabetes mellitus also suffered more frequently from *Candida* superinfection, although the difference was not statistically significant (*p* = 0.070) (Table 3).

**Table 3:** Comparison of predisposing factors for *Candida* superinfection in OLP

Influencing factors	No infection (*n* = 171)	Superinfection (*n* = 97)	*P *^a^
	No. (%)	No. (%)	
Demography			
Mean age (SD)	61.7 (± 12.8)	67.6 (± 10.1)	< 0.001
Gender (male)	39 (22.8)	19 (19.6)	0.539
Local			
Erosive OLP	63 (36.8)	49 (50.5)	0.029
Topical steroids	27 (15.8)	15 (15.5)	0.944
Inhaled steroids	7 (4.1)	7 (7.3)	0.271
Removable denture	33 (19.5)	30 (31.3)	0.031
Systemic			
Hypothyroidism	47 (27.8)	29 (29.9)	0.717
Diabetes mellitus	18 (10.7)	18 (18.6)	0.070
Autoimmune disease	22 (13.0)	22 (22.7)	0.041
Asthma/COPD	15 (8.9)	13 (13.4)	0.247
Drug-induced ^b^			
Antihypertensives	77 (45.6)	63 (65.6)	0.002
Psychotropic drugs	12 (7.1)	19 (19.8)	0.002
Proton pump inhibitors	14 (8.3)	12 (12.5)	0.267
Lifestyle			
Smoker	27 (16.0)	13 (13.5)	0.595
Former smoker	33 (19.5)	22 (22.9)	0.513

Complete data for concomitant diseases were available from 266 patients and for medication, presence of removable dentures and smoking from 265 patients

*SD* standard deviation; *OLP* oral lichen planus; *COPD* chronic obstructive pulmonary disease

^a ^No infection vs. superinfection

^b ^Drugs with a high level of evidence for causing hyposalivation

The binomial logistic regression analysis, including the factors mentioned above, revealed that only two variables were statistically significant: age (*p* = 0.016) and the intake of psychotropic drugs (*p* = 0.008). Each year of life increased the odds of contracting *Candida* superinfection by OR = 1.03 (95% CI [1.01, 1.06]), as did intake of psychotropic drugs (OR = 3.15 95% CI [1.36, 7.29]). The logistic regression model was statistically significant, χ^2^(6) = 28.19, *p* < 0.001. The Hosmer–Lemeshow test indicated a good model fit (χ^2^(8) = 4.96, *p* = 0.762). Correlations between predictor variables were low (Pearson correlation r < 0.40), indicating that multicollinearity was not a confounding factor in the analysis.

## Discussion

In the Department of Periodontology, Oral Medicine and Oral Surgery of the Charité - Medical University Berlin, oral swabs are routinely used for the detection of *Candida* species in cases of suspected infections/superinfections. The advantages of this method are the site-specific isolation of viable cells and the easy application of this diagnostic tool. The disadvantage of this method is that it does not allow quantification but only a semi-quantitative estimation of microorganisms [20]. The most reliable diagnosis of *Candida* infection is the detection of hyphae invading the tissue [21]. Nevertheless, the oral swab technique was used because the focus of this research was on the correct identification of *Candida* species and the clinical relevance of oral fungal infections in OLP.

Regarding the species spectrum, *C. albicans* was the predominant species in OLP patients. *Non-albicans* species accounted for 27% in total. This is consistent with recent findings by *Arora* and colleagues who also found a prevalence of 27% of *non-albicans* species in OLP patients in their prospective study [14]. Earlier studies reported a lower proportion of *non-albicans* species in OLP patients [10, 15, 22, 23]. This discrepancy may already be due to an emerging selective process or may be caused by differences in sample collection and methods for species identification. In agreement with other reports, the most frequently isolated *non-albicans* species was *C. glabrata* [24, 25]. *C. dubliniensis* was the second most common *non-albicans* species in the present study. Although this species was first detected in the oral cavity of HIV-positive patients, several studies have reported the detection of *C. dubliniensis* in HIV-negative patients and healthy persons [26, 27]. In the present study, none of the included patients was HIV positive. In recent years, *C. dubliniensis* has also been detected in the oral cavity of OLP patients, with and without oral candidiasis [14, 28–30]. These data and the present findings confirm the assumption that *C. dubliniensis* may also be a part of the oral microflora of patients with OLP.

Many local and systemic factors have been previously identified to enhance oral carriage of *Candida* species. These factors include, but not limited to, use of dentures, local steroids, tobacco smoking, reduced salivary flow, immunosuppressed states (such as HIV or secondary to age), endocrine disorders, receipt of xenogeneic drugs and broad-spectrum antibiotics [31–35]. In the present study, a number of such factors were analysed. To take all these factors into account, it was necessary to conduct multivariate analyses using binomial logistic regression.

So far, no study of patients with OLP has assessed the influence of certain factors on the composition of the yeast spectrum. Thus, the primary aim of this study was to identify factors associated with the detection of *C. dubliniensis* and other *non-albicans* species, respectively, compared to *C. albicans*. As a result, logistic regression analysis indicated a significant association between tobacco smoking and the presence of *C. dubliniensis.* Recent evidence supports these findings, as this study demonstrated that exposure of oral *C. dubliniensis* isolates to cigarette smoke condensate significantly enhanced in vitro adhesion traits and haemolysin production of these isolates [36]. All these attributes are considered essential virulence factors of *Candida* species, thereby increasing the pathogenicity of this species in the presence of cigarette smoke. Although *C. albicans* isolates showed a comparable increase of pathogenic attributes in the abovementioned study, in our investigation tobacco smoking was significantly more prevalent in *C. dubliniensis* carriers than in *C. albicans* carriers (*p* < 0.001). Smoking further reduces gingival exudate, resulting in a decrease in the number of leukocytes and immunoglobulins in the oral cavity [37]. Both are important factors in host defense to prevent *Candida* colonization [31]. In accordance with research by Al-Karawii et al., who reported local colonization-enhancing factors in 93% of the patients carrying *C. dubliniensis*, in the present study 91% of the patients carrying *C. dubliniensis* exhibited those factors that may enhance colonization/infection by this species [28]. Kragelund et al. found this species to be overrepresented among OLP patients who were previously exposed to antimycotic drugs [30]. Since data regarding antimycotic treatment was only available between 2015 and 2019, it was not possible to verify the findings of Kragelund and coworkers in our study population. In conclusion, the diversity of the available data indicates a high variance in the epidemiological profile of *C. dubliniensis,* and further studies will be necessary to clarify its distribution pattern as well as the clinical implication of the presence of this yeast species.

Previous studies have reported high prevalences of *non-albicans* species in oral specimens of denture wearers with or without denture stomatitis [38–40]. Consistent with this, logistic regression analysis in this series revealed that using removable dentures was significantly associated with the presence of *non-albicans* species (other than *C. dubliniensis*). It has been hypothesized that the frequent detection of *non-albicans* species in denture wearers may already reflect a selection process due to multiple antifungal treatments [40]. It has further been demonstrated that *non-albicans* species have a high cell surface hydrophobicity and a distinct ability to form biofilms compared to *C. albicans*, traits found to be beneficial for adherence to acrylic surfaces [25, 41, 42]. In addition, the mean age of patients carrying *non-albicans* species was higher than that of patients carrying only *C. albicans*, although this association was not significant. Nevertheless, this trend was probable, as the frequency of denture use increases with age.

The secondary aim of the study was to determine independent factors that increase the risk of superinfection of OLP lesions with *Candida* species. Multivariate analysis identified increasing age and the use of psychotropic drugs as independent risk factors for *Candida* superinfection in the present cohort studied. Matching these findings, low unstimulated salivary flow rate and the intake of anxiolytics have already been found to be risk factors for oral fungal infections in OLP subjects [43]. Moreover, various psychotropic drugs such as antidepressants, antipsychotics, hypnotics and benzodiazepines, but also antiepileptic drugs (especially GABA agonists) show a high level of evidence for causing hyposalivation as an adverse drug reaction [44, 45]. In a large-scale study with more than 1200 participants, it was found that both increasing age and the use of psychotropic drugs were significantly associated with subjective dry mouth and an unstimulated salivary flow rate < 1 ml/min [46]. Reduced salivary flow rate, which may be age related, drug induced or due to an underlying disease, is a known predisposing factor for oral candidiasis [31]. Furthermore, it has been shown that the prevalence of oral candidiasis increases with age, although it is not clear whether age per se is a predisposing factor [47]. However, the present study was able to show through multivariate analysis that the likelihood of contracting *Candida* superinfection increased with each year of life.

Extrapolation of the study results may be affected by several limitations. Firstly, due to the retrospective nature of the present study, it was not conducted under controlled conditions and there is a possibility of missing data. This fact in mind and due to the small sample size of patients carrying *C. dubliniensis*, the results of this study should be understood as a trend. Furthermore, in this retrospective analysis neither *Candida* quantification nor testing of susceptibility to common antifungal drugs could be performed. Future and prospective studies might consider these aspects.

## Conclusion

The results of this study indicate that *C. albicans* is the predominant species in OLP patients, but *non-albicans* species account for over 27% of all isolates. In smoking patients and subjects with removable dentures, *C. dubliniensis* and other *non-albicans* species were significantly more frequently detected than in non-smokers and patients without removable prostheses. Despite the limitations of the study, this should be considered in the antimycotic therapy of superinfected OLP lesions. In particular the use of azole antifungals in patients matching the criteria mentioned above should be conducted restrainedly due to potential drug resistance. Hence, yeast identification prior to antimycotic therapy appears to be recommendable, especially as resistance testing is usually not performed for oral candidiasis treatment. Furthermore, patients of increasing age and/or on psychotropic drugs require special attention in order to initiate appropriate therapy, i.e. early administration of antimycotic drugs.
